# Primaquine plus artemisinin combination therapy for reduction of malaria transmission: promise and risk

**DOI:** 10.1186/s12916-016-0611-9

**Published:** 2016-04-01

**Authors:** Chandy C. John

**Affiliations:** Ryan White Center for Pediatric Infectious Disease and Global Health, Indiana University School of Medicine, 1044 W. Walnut St., R4 402C, Indianapolis, IN 46202 USA

**Keywords:** Malaria, Falciparum, Artemisinin combination therapy, Artemether-lumefantrine, Primaquine, Gametocyte, Transmission, Mass drug administration, Membrane feeding assay, Hemolysis

## Abstract

Reduction of gametocyte transmission from humans to mosquitoes is a key component of malaria elimination. The study by Gonçalves and colleagues provides valuable new data on how the addition of low-dose primaquine to artemether-lumefantrine affects reduction of gametocytemia and transmission of gametocytes to mosquitoes in asymptomatically *Plasmodium falciparum*-infected children without G6PD deficiency, and on the degree to which low-dose primaquine affects hemoglobin levels in these children. The study sets the stage for future research required for consideration of an artemisinin combination therapy (ACT)-primaquine regimen in mass drug administration campaigns. Future studies will need to evaluate toxicity in adults and G6PD deficient persons, assess gametocyte transmission from adults, evaluate different ACT drugs with primaquine, and assess the implications of “rare” toxicities in large treatment populations, such as hemolysis requiring blood transfusion. The study highlights both the promise and the potential risk of ACT-primaquine treatment in malaria elimination campaigns.

Please see related article: https://bmcmedicine.biomedcentral.com/articles/10.1186/s12916-016-0581-y.

## Background

### Primaquine + artemisinin combination therapy (ACT) for malaria elimination

As countries consider campaigns to eliminate malaria, the question of gametocyte transmission from the human to mosquito host takes center stage: without reduction of gametocyte transmission to low or zero levels, it will be hard to achieve malaria elimination. Artemether-lumefantrine (AL), the most common first-line artemisinin combination therapy (ACT) for treatment of malaria in Africa, reduces gametocytemia and gametocyte transmission to mosquitoes, but does so less effectively than primaquine. Current World Health Organization (WHO) guidelines state that “in elimination areas which have not yet adopted primaquine as a gametocytocide for *P. falciparum* malaria, a single 0.25 mg base/kg primaquine dose should be given to all patients with parasitologically-confirmed *P. falciparum* malaria on the first day of treatment in addition to an ACT, except for pregnant women and infants < 1 year of age” [1]. Most studies of children with symptomatic malaria treated with ACT alone show that, regardless of the ACT used, a small percentage of children continue to transmit gametocytes to mosquitoes 7–14 days after treatment [2–4]. However, similar studies in children with asymptomatic parasitemia are lacking, and there is little data on how the addition of primaquine to ACT might alter gametocyte transmission in this population. A key issue with the addition of primaquine to ACT is the risk of hemolysis with primaquine, a risk that is substantially increased in individuals with glucose-6-phosphate dehydrogenase (G6PD) deficiency, a condition common in sub-Saharan Africa.

Gonçalves et al. address this gap through a well-designed and carefully conducted study in which they assessed the efficacy of AL, AL plus 0.25 mg/kg of primaquine, or AL plus 0.40 mg/kg of primaquine on reduction of gametocytemia and gametocyte transmission in 360 children in Burkina Faso who were asymptomatically infected with *Plasmodium falciparum* [5]. Screening for G6PD deficiency was done with BinaxNOW rapid testing, and only children without G6PD deficiency were enrolled in the study. The addition of primaquine to AL significantly reduced the prevalence of gametocytemia. However, though prevalence of gametocytemia as measured by Pfs25 quantitative nucleic acid sequence-based amplification (QT-NASBA) was still 10–42 % in the different study arms 14 days after treatment initiation, only one child (in the AL alone arm) transmitted gametocytes to mosquitoes in the 3 to 14 days after treatment initiation. The study findings suggest that gametocyte transmission to mosquitoes from children with asymptomatic parasitemia is lower than in symptomatic children, and that AL or AL plus primaquine at either dose all substantially reduce gametocyte transmission from asymptomatically infected children to mosquitoes. Two children had a hemoglobin decrease of > 4 g/dL, and both were on primaquine treatment (one at 0.4 mg/kg, one at 0.25 mg/kg). The study provides valuable new data that enhances our understanding of the additive effects of low-dose primaquine to AL on gametocytemia and on transmission of gametocytes, but also highlights the need for additional studies on the role of primaquine supplementation to ACT in reducing malaria transmission.

### Infectivity in asymptomatic vs. symptomatic infection, and in children vs. adults

For example, further studies are needed to define who should be treated with an ACT-primaquine combination for malaria elimination: asymptomatic individuals in a mass screen and treat or mass drug administration (MDA) program, only symptomatic individuals who seek treatment, or both symptomatic and asymptomatic individuals? In areas of unstable transmission, rates of asymptomatic gametocytemia may remain high during the low malaria transmission season, so the population can easily transmit gametocytes to mosquitoes when the mosquito population increases [6]. In these areas, MDA to asymptomatic individuals in the off-peak season will be critical. However, some areas with very low transmission, such as the Nandi Hills highland areas of Kenya where we have conducted studies, appear to have little to no asymptomatic parasitemia or gametocytemia during low malaria transmission periods [7]. In these areas, rapid treatment of symptomatic individuals with ACT-primaquine combinations or possibly seasonal malaria chemoprophylaxis may be more effective to reduce malaria transmission or incidence than an MDA campaign that treats few or no infected individuals.

In addition, most studies that have assessed gametocyte transmission have assessed transmission from infected children [2, 4, 8], but infected adults can also transmit gametocytes. Studies of the effect on ACT-primaquine combinations on reduction of gametocyte transmission from adults are needed, because MDA campaigns aiming for malaria elimination will target children and adults. Also, as the authors point out, the membrane feeding assays they and others have used underestimate human to mosquito gametocyte transmission when compared to superior but impractical direct mosquito feeding assays. Studies using membrane feeding may therefore overestimate the efficacy of, for example, AL alone in reducing gametocyte transmission. Models that account for this underestimation may be required for accurate planning of MDA campaigns.

### ACT: the complex effects and interactions of the partner drug

The long half-life of piperaquine may make dihydroartemisinin-piperaquine (DP) the ACT of choice for MDA campaigns. Modeling studies also suggest that in elimination campaigns primaquine is most effective when partnered with a long-lasting prophylactic drug like DP [9]. However, DP appears to be less effective than AL in reducing gametocyte transmission to mosquitoes in children with uncomplicated malaria [4]. It is possible that the addition of primaquine may therefore be more important in MDA elimination campaigns using DP than AL, because primaquine may be more important for reduction of gametocyte transmission in individuals treated with DP as opposed to AL. Hematologic toxicities with piperaquine as compared to lumefantrine as the ACT partner drug are not yet known. A recent study showed that primaquine does decrease gametocyte transmission when added to DP as treatment for uncomplicated malaria [10]. The investigators in the present study are currently pursuing the logical next study, assessing the effect of DP plus primaquine on gametocyte transmission in asymptomatically infected children in the Gambia [11]. The results of this study will be important to assessments of the feasibility and safety of MDA programs with DP-primaquine combinations.

### Population-level toxicity: primaquine-related hemolysis with or without G6PD deficiency

Ultimately, however, the place of primaquine in addition to ACT in MDA campaigns depends not only on its efficacy in reducing gametocyte transmission but also on its safety in MDA programs. The primary safety concern with primaquine treatment is hemolysis and consequent anemia. Primaquine was used safely in MDA programs outside Africa for *P. vivax* elimination [12], but the prevalence of G6PD deficiency and anemia in those countries differs greatly from that in African countries. In a study in Tanzania, Shekalaghe et al. found that primaquine at 0.75 mg/kg combined with sulfadoxine-pyrimethamine (SP) and artesunate led to a significant reduction in hemoglobin in children when compared to SP plus artesunate alone [13]. Decreases in hemoglobin were greatest in children with G6PD deficiency, but also occurred in children without G6PD deficiency (as defined by genetic testing). In the study by Gonçalves et al., decreases in hemoglobin were greater with primaquine treatment, though the differences reach statistical significance only for the 0.4 mg/kg primaquine arm [5]. A hemoglobin decrease of > 4 g/dL occurred in 1 of 75 children (1.3 %) and 1 of 73 children (1.4 %) in the 0.4 mg/kg and 0.25 mg/kg primaquine arms, respectively. Hemoglobin levels recovered by the end of the study (apparently without intervention), and no child required a blood transfusion.

However, the possibility that ~ 1 % of children in a low-dose primaquine MDA program may have a > 4 g/dL decrease in hemoglobin raises significant concerns. In the study by Gonçalves et al, only children with a hemoglobin level ≥ 8/dL who were not G6PD deficient were eligible for participation [5]. Risks of hemolysis in children with G6PD deficiency will be substantially higher. Before MDA campaigns with low-dose primaquine are considered, assessment of safety in G6PD deficient African children is imperative, because accurate testing for G6PD deficiency in mass campaigns will likely not be feasible. Furthermore, will MDA campaigns be able to screen for anemia and exclude anemic children? How many of those anemic children will be gametocytemic? How many serious adverse events will occur if ~ 1 % of children have a hemoglobin drop of > 4 g/dL? How many children will have a decrease in hemoglobin that requires blood transfusion? Even a very low 0.1 % incidence rate will result in 100 such events in a population of 100,000 children. With the known lack of capacity for timely blood transfusion in many areas of Africa, such numbers should lead to very careful assessment about the possibility of severe morbidity or even mortality as a result of ACT plus low-dose primaquine MDA.

## Conclusions

The addition of primaquine to ACT could be an important tool in malaria elimination campaigns. The study by Gonçalves et al. has provided new information that highlights both the promise and the potential peril of adding primaquine to ACT in children with asymptomatic parasitemia. The study also helps to highlight a number of questions centered on transmission and safety that must be answered prior to consideration of this combination for MDA. The promise of this therapy is too great to neglect based on theoretical concerns about toxicity, yet the risks of implementation without a thorough assessment of safety are equally great. Serious adverse events, even in a small number of children, could set back MDA efforts for years. So onward to the next tier of studies, with the goal of determining a successful combination that is both safe and effective.
